# Business Brand Research Based on Multi-Feature Fusion Emotion Analysis

**DOI:** 10.3389/fpsyg.2022.939304

**Published:** 2022-09-27

**Authors:** Boxuan Li

**Affiliations:** College of Foreign Languages, Jilin University, Changchun, China

**Keywords:** multi-feature fusion, emotional analysis, commercial brand, supplements, consumers

## Abstract

With the deepening of globalization, brand plays an important role in determining the competitiveness of enterprises. It is worth thinking about how to quantify the brand value reasonably to achieve the purpose of improving the competitiveness of enterprises. The research of commercial brands based on emotion analysis extracts the views of consumers on the evaluation data of brand attributes, analyzes the emotional tendency of consumers' views, and then helps enterprises adjust their production strategies. The purpose of emotion analysis is to judge users' views and attitudes toward goods or services by analyzing texts. In this paper, the linear support vector machine model is used to extract and study the features in sentiment analysis. Then, the brand based on the fusion model is evaluated, and the experimental conclusions are drawn: the method of fusing the depth features extracted by word vector and the named entity extracted by CRF makes the brand effect of fusing emotional factors better than the feature extraction method only using CRF named entity recognition. It is proved that the model method proposed in this paper has a certain role in actual business. Feature fusion often combines shallow model methods and fuses various shallow feature screening technologies based on word segmentation results. This paper introduces fusion features and supplements feature vectors based on the results of the deep learning word vector model. Support Vector Machine (SVM) maps feature vectors to some sample points in the space, finds a plane that can realize sample classification, and maximizes the distance between the two sample points closest to the plane in two types of samples, to maximize the classification performance and improve the generalization of the model.

## Introduction

Emotion is the embodiment of a person's inner feelings, and emotional analysis ability is one of the symbols of EQ. In this paper, we propose and compare multi-feature-based emotional state recognition algorithms and their differences. Features of different emotions on the same day tend to gather more closely than those of the same emotions on different days (Picard et al., 2001). In this paper, a new multi-feature-based emotion recognition method is proposed and experiments are carried out. Experiments show that this method can accurately analyze emotions, including happiness, sadness, anger, and so on (Zhao et al., 2004). Detecting relevant opinions and emotions in text data is the focus of emotion analysis. We propose a new tool, EmoWeb, which is a prototype of a dynamic emotion analysis tool, including a visual and dynamic framework for analyzing text, which is based on a dictionary of multi-feature emotion analysis (Isaac et al., 2018). In this paper, the text sentiment analysis technology is used to deeply mine the text information of online famous brand reviews, to reflect the attributes and attitudes of consumers. Grab the mobile phone review data and search engine index for verification, to show the online word-of-mouth of commercial brands, consumers' attention to various attributes of goods, emotional attitudes, and so on (Yan et al., 2021). Local commercial brands are regarded as tools of birthplace image. Using the best famous brands to promote themselves has limitations, which are only applicable to the country, but not to smaller places. This paper presents the image relationship between local brands and commercial brands originating from that place. A place, as well as local brands, needs to define its image competition and strength areas. In this paper, Need Scope technology is used to analyze the degree of competition or conflict between commercial brands and Poznan city brand image (Florek, 2013). This paper discusses the connotation and value of the commercial brand and holds that commercial brand competition is the general trend of commercial development. Merchants should consolidate the construction of retail brands with modern concepts, change the mode of economic growth, improve the core competitiveness, and implement the competitive strategy of commercial brands (Hong, 2007). The sustainability of commercial brands has become a major problem for enterprises. The purpose of this paper is to explore the influence of brand image and brand loyalty on brand equity. Exploratory factor analysis and confirmatory factor analysis are used for causality analysis and fitting analysis. The results show that brand image and brand loyalty have a strong influence on brand equity (Azad et al., 2013). Brand competitiveness contributes to the development of enterprises. This paper expounds the relevant theories of commercial brand competitiveness and puts forward a variety of solutions for how to enhance brand competitiveness: 1. Study core products, 2. Create brand culture, 3. Maintain good service attitude, and 4. Maintain innovation ability. The effectiveness and practicability of this method are proved by the empirical application of many enterprises, which provides a new idea for the development of commercial brands (Xiong and Cheng, 2020). Today, with the rapid economic development, most brands in China are still manufacturers' brands. Compared with other countries, our commercial brands still need to be developed. This paper discusses the present situation of the development of commercial brands at home and abroad, and the significance and measures of implementing commercial brand strategy (Xu, 2003). This paper analyzes the essential characteristics of commerce and studies the relationship among commerce, brand, and consumption. This paper puts forward the position and function of brand consumption culture in modern commerce. Commerce is more faced with brand consumption culture, while the brand system shows that the essence of design is to influence, transform, or create a new fashion and ideal lifestyle (Wei and Ouyang, 2003). The purpose of this paper is to investigate people's use and satisfaction with the commercial brand kimchi. A questionnaire survey was adopted, and the results showed that nearly half of the people who bought commercial brand kimchi thought that their taste was the main reason for buying commercial brand kimchi. Consumers of commercial brand kimchi have high satisfaction with kimchi packaging, while their price is the factor of low satisfaction (Kim and Ryu, 2007). At present, there are still many problems in the construction of commercial brands in China, such as the lack of well-known brands and international brands, and the lack of correct cognition of commercial brand construction. These problems restrict the development of commercial brand building in China. We should further guide and establish brand awareness, support the construction of commercial brands, and create a good environment for the construction of commercial brands (Yang, 2017). The brand is an important visual factor, which can effectively identify business names and highlight the differences from other competitors. This paper studies the role of brand in the business name and customer satisfaction and loyalty. For this reason, this study randomly selected 380 dairy consumers in Korea as samples and demonstrated the causal relationship among brand awareness, business name, and customer satisfaction through the intermediary role of brand interests (Askari et al., 2013). Through questionnaire survey and mathematical statistics, this paper studies the influence of demographic variables on brand loyalty of commercial fitness clubs, and analyzes that consumers have great differences in brand awareness and loyalty under the background of different genders, ages, and incomes (Shen and Qingshan, 2015). This paper studies the strategic competition of a city's commercial brand. Brand strategy is not only to create a well-known brand but also to distinguish itself from other competitors and attract consumers. Residents and investors are stakeholders in the city brand goal, and it is very important for investors and entrepreneurs to develop a city brand image (Kaya and Marangoz, 2014).

## Commercial Brand Analysis

### The Definition of the Brand

The consumer awareness of products and product lines is an intangible asset. The connotation and essence of the brand are its value, culture, and personality. The meaning of brand mainly includes the following three aspects: First, brand interests: brand represents the attributes of specific goods, and brand not only represents a series of attributes but also brings functional or emotional benefits to consumers. The second is the value culture: the brand also reflects some values of its manufacturers, and the brand also has a specific culture. The third is personality: the brand reflects some personalities of manufacturers and customers, which are often valued by consumers, followed by the types of consumers who buy or use the product.

### The History of the Brand

The word brand dates back to Old Norse, from which the modern Scandinavian language originates. A brand originally refers to a burning piece of wood. It was not used as a verb until the late middle Ages when it meant “permanently marked with hot iron.” By the seventeenth century, it was a sign of ownership of items. Stone Age cave paintings suggest that early people might mark cattle with symbols in paint and tar. By 2000 BC, livestock owners used a more permanent method to use a burn mark. Some of the earliest known markers of Chinese pottery date from 4000 to 5000 years. Some trademarks of animal seals and number symbols appeared in India as early as 2600 BC. In Athens, the commercial center of ancient Greece, cosmetic brands appeared in advertising poems, and with the emergence of ancient Egyptian characters, there was a trend of brand and advertising development. In the Middle Ages, paper makers distinguished themselves from other manufacturers by using watermark marks and Renaissance artists marked their works in new ways, replacing symbols with signatures. After the Industrial revolution, factories borrowed winemakers to print logos on barrels because of productivity improvements and large-scale manufacturing of various commodities, which made similar products uncompetitive. Soon there were popular brands such as Campbell's Soup and Coca-Cola.

### Commercial Brand Concept

The so-called commercial brand refers to the commercial circulation brand, generally referred to as the commercial brand, which refers to the commercial enterprise brand, commodity brand, service brand, employer brand, and financial brand that stand out from the market competition, are recognized by the public, are protected, and can produce huge effect under the condition of the market economy.

### Modern Business Brand

The original intention of some modern commercial brands in the pursuit of cooperation with artists is to make their own brands “artistic” and integrate artists and artworks into their brand value. The most ideal “art” is that the whole brand is an “artist” and the product is the theme of the art brand. The theme, appearance, marketing, advertising and stores of commodities all have artistic participation. Consumers no longer only pay attention to the brand, but begin to pursue the sense of design and art of commodities. Exquisiteness, exclusivity, individuality and emotional resonance become the goals pursued by commodities.

### Brand Communication

Brand communication is an important link to play the enterprise brand influence and promote the enterprise development. It is not only related to the market competitiveness of enterprise products but also related to customer loyalty and enterprise reputation. The heavy task of brand image communication is placed on microfilms, which not only fit the characteristics of audience receiving information fragmentation in the new media era but also have the advantages of artistic, entertainment, and story. At the same time, they have their unique advantages in low-cost production and advertising. Generally speaking, the surface factors of brand communication are name, image, color, packaging and other information content is limited. Product characteristics, benefits, service commitments, brand awareness, brand association and other deep-seated factors aggregate more abundant information, which is the source of brand communication information and determines the aggregation of brand communication information. In addition, the information sources of these brand communication also have corresponding performance in the communication process of microfilms, first manifested as the brand cognition, which is mainly obtained through the audience clicking to play microfilms, then the emotion or attitude toward the brand, mainly through the audience evaluation in the process of watching.

### The Relationship Between the Visual Image of a Commercial Brand and Consumer Perception

The visual image of commercial brands conveys the brand concept to consumers in the form of a portrait, which can help commercial brands to establish a more high-quality and strong brand image in the eyes of consumers. A good brand visual image can bring a pleasant aesthetic to customers, show the innovation ability of the enterprise, and build a bridge between enterprises and consumers while attracting consumers. The visual image of commercial brands also includes the brand logo, brand product packaging, and other contents, which are spread by advertising, oral communication (language expression), and network, so that consumers can have a certain perception of the brand image. When promoting commercial brands, their visual image needs to grasp the factors, establish a good perceptual impression and social foundation through consumer feedback, and maintain consumers' sense of trust and loyalty to the brand.

## The SVM Model Algorithm

### Linear Optimum Hyperplane

In statistical learning, VC dimension means that for a given data set with N samples, this data set has 2^*N*^ classification methods. If a given decision function set can classify all cases, the VC dimension of the function set is called N. It can reflect the generalization ability of the model. The most important index to measure the algorithm is generalization ability. The stronger its ability, the higher the performance of the model on the test set. The generalization ability of the algorithm is defined as follows, as shown in Formulas 1, 2.


(1) $$\begin{array}{l} R\left(\alpha \right)\leq R_{emp}\left(\alpha \right)+\psi \left(\frac{N}{h}\right) \end{array}$$

(2) $$\begin{array}{l} \psi \left(\frac{N}{h}\right)=\sqrt{\frac{h\left(\ln\;\left(\frac{2N}{h}\right)\right)-\ln\;\left(\frac{\eta }{4}\right)}{N}} \end{array}$$

Where ψ(Nh) is called the confidence range, h is the VC dimension, and N is the number of samples. Structural risk minimization of the SVM is achieved by optimizing the superlayer. Given a hyperplane, the *w*^*T*^*x* + *b* = 0. If the hyperplane can satisfy Formula 3,


(3) $$\begin{array}{l} y=\{\begin{array}{c} 1,w^{T}x+b\geq 1\\ -1,w^{T}x+b\leq -1 \end{array} \end{array}$$

Training samples can be divided into two categories, so we call this problem linearly separable. Let the interval between hyperplanes *w*^*T*^*x* + *b* = 1 and *w*^*T*^*x* + *b* = −1 be Δ. Then, the hyperplane is an Δ interval classification hyperplane. The relationships are shown in Formula 4.


(4) $$\begin{array}{l} h\leq \min\left(\left[\frac{R^{2}}{\Delta^{2}},n\right]\right)+1 \end{array}$$

Where n is the number of dimensions of the sample, R means that all samples are surrounded by a sphere of radius R. The above formula shows that in a certain range, the interval is inversely proportional to the size of the VC dimension, which can improve the generalization ability of the model by increasing the classification interval.

### Linear Support Vector Machine

The concept of soft interval classification hyperplane is introduced when the linear support vector machine is used to solve the problem of linear inseparable of the number training data set. This is a classification hyperplane with a small number of samples that are not divided correctly. After ignoring the wrong classification sample, Formulas 5, 6 can be used to represent the optimization target—the large classification interval objective function of the soft interval classification hyperplane.


(5) $$\begin{array}{l} l_{1}:w^{T}x+b=1 \end{array}$$

(6) $$\begin{array}{l} l_{2}:w^{T}x+b=-1 \end{array}$$

Then, the expression of the classification hyperplane in the middle is *w*^*T*^*x* + *b* = 0. Let *x*_*i*_ and *x*_*j*_ be any point on *l*_1_ and *l*_2_, then the distance G between *l*_1_ and *l*_2_ is the projection of the connecting line segment Δ to the normal vector *w*. Equation 7,


(7) $$\begin{array}{l} d=w^{T}\left(x_{i}-x_{j}\right)/\|w\| \end{array}$$

Formula 8 by combining Formulas 5, 6,


(8) $$\begin{array}{l} d=\frac{2}{\left\|w\right\|} \end{array}$$

Turning the non-linear classification problem into a linear separation problem not only requires a soft interval but also one solution to introduce the kernel function. That is, to map the nonlinear sample data points in the original low-dimensional space that is not high to the high-dimensional feature space. Then, in a high-dimensional space, the support vector machine method is used to solve the classification hyperplane for classification. Nonlinear problem requires a nonlinear mapping first to make the original space mapping into a new and higher dimensional feature space. In a linear separable problem, the size of the expected risk is determined by the confidence interval, which is the lower expected risk. As shown in Formula 9.


(9) $$\begin{array}{l} \underset{w,b}{\max}\frac{2}{\left\|w\right\|} \end{array}$$

Converts the target problem to a convex form of the optimization problem, as shown in Formula 10.


(10) $$\begin{array}{l} \underset{w,b}{\max}\frac{{\left\|w\right\|}^{2}}{2} \end{array}$$

The Lagrangian function can be obtained by introducing the Lagrangian dual multiplier α, as shown in Formula 11.


(11) $$\begin{array}{l} L\left(w,b,\alpha \right)=\frac{1}{2}{\left\|w\right\|}^{2}+\overset{N}{\underset{i=1}{\sum }}\alpha_{i}\left(1-y_{i}\left(w^{T}x_{i}+b\right)\right) \end{array}$$

The Lagrange function is a function that only has conservative force in the mechanics department and describes the dynamic state of the whole physical system.

Formulas 12, 13 can be obtained by calculating partial derivatives of *w* and b.


(12) $$\begin{array}{l} \frac{\partial L}{\partial w}=w-\overset{N}{\underset{i=1}{\sum }}\alpha_{i}y_{i}x_{i}=0 \end{array}$$

(13) $$\begin{array}{l} \frac{\partial L}{\partial b}=\overset{N}{\underset{i=1}{\sum }}\alpha_{i}y_{i}=0 \end{array}$$

The substitution of Formulas 12, 13 gives us the dual problem of the original equation, as shown in Formula 14.


(14) $$\begin{array}{l} \underset{\alpha }{\max}\left(\overset{N}{\underset{i=1}{\sum }}\alpha_{i}-\frac{1}{2}\overset{N}{\underset{i,j=1}{\sum }}\alpha_{i}\alpha_{j}y_{i}y_{j}x_{i}x_{j}\right) \end{array}$$

Assuming α^*^ is the optimal solution of the dual model, the normal vector of the obtained decision equation is *w* $=\overset{N}{\underset{i=1}{\sum }}\alpha_{i}^{*}y_{i}x_{i}$, the offset is b^*^ $=y_{j}-w^{T}x_{j}j\in \left\{j|0<\alpha_{j}^{*}\right\}$, and the final decision function is *f*(*x*)=*sgn*(*w*^*T*^ * *x* + b^*^).

The support vector machine method is very elegant and complete mathematically, but there are many clever implementation methods in computer programming, such as using SMO algorithm to solve the dual problem. At the same time, the support vector machine is widely used, and there are many mature implementations, which are very suitable for the basic control algorithm. MO is the abbreviation of Sequential Minimum Optimization Algorithm, which is used to solve the optimization problems in the training process of support vector machines. SMO was invented by John Pratt of the Microsoft Research Institute in 1998. It is widely used in the training process of SVM and implemented in the popular SVM library LIBSVM.

### Kernel Function

In the nonlinear case, it is transformed into a dual equation of the target equation. The purpose of turning to the dual equation is to make the relationship between the samples of the target equation become an internal product, which is easy to generalize to the non-linear classification problems later. When the linearity is not separable, it is not necessarily possible, so the training samples are distributed on both sides, making the empirical risk zero. The relaxation variable can then be introduced, which represents the extent to which the training sample can be tolerated by the “error.” The constraint change to $y_{i}\left(w^{T}x_{i}+b\right)\geq 1-\xi$, and the sum of the degree of the “wrong” partition is $\overset{N}{\underset{i=1}{\sum }}\xi_{i}$. The effective method to solve the linear separation of support vector machine is to ensure the maximum gap and small mismatch, with the target Equation 15.


(15) $$\begin{array}{l} \underset{w,b}{\min}\frac{{\left\|w\right\|}^{2}}{2}+C\overset{N}{\underset{i=1}{\sum }}\xi_{i} \end{array}$$

Where C is the penalty coefficient. After introducing Lagrange multipliers α_*i*_ and β_*i*_, the Lagrange equation is obtained as shown in Formula 16.


(16) $$\begin{array}{l} L\left(w,b,\alpha ,\beta \right)=\frac{1}{2}{\left\|w\right\|}^{2}\\ +\overset{N}{\underset{i=1}{\sum }}\alpha_{i}\left(1-y_{i}\left(w^{T}x_{i}+b\right)\right)+C\overset{N}{\underset{i=1}{\sum }}\xi_{i}-\overset{N}{\underset{i=1}{\sum }}\beta_{i}\xi_{i} \end{array}$$

The most basic support vector machine model can only deal with the case that the data is linearly separable, and its basic principle is quite simple, but the solution and optimization of the model are very complex. The conventional way is to take the non-negative relaxation variables as partial derivatives of *w*, *b*, and ξ_*i*_, and obtain Formulas 17–19.


(17) $$\begin{array}{l} \frac{\partial L}{\partial w}=w-\overset{N}{\underset{i=1}{\sum }}\alpha_{i}y_{i}x_{i}=0 \end{array}$$

(18) $$\begin{array}{l} \frac{\partial L}{\partial b}=\overset{N}{\underset{i=1}{\sum }}\alpha_{i}y_{i}=0 \end{array}$$

(19) $$\begin{array}{l} \frac{\partial L}{\partial \xi_{i}}=C-\alpha_{i}-\beta_{i}=0 \end{array}$$

Replacing Formulas 17–19 into Formula 16 obtains the dual form of the original equation, as shown in Formula 20.


(20) $$\begin{array}{l} \underset{\alpha }{\max}\left(\overset{N}{\underset{i=1}{\sum }}\alpha_{i}-\frac{1}{2}\overset{N}{\underset{i,j=1}{\sum }}\alpha_{i}\alpha_{j}y_{i}y_{j}x_{i}x_{j}\right) \end{array}$$

The SVM belongs to a generalized linear classifier. They can also be considered as a special case of the Tikloff normalization (Tikhonov Regularization) method. This family of classifiers features that they can simultaneously minimize the empirical error and maximize the geometric edge zone, so the SVM is also known as the maximum edge zone classifier. It maps the original sample space to the nonlinear feature space in the higher dimension, which was originally non-linear separable in the low dimension, and becomes a linear separable problem.

### Non-Linear Support Vector Machine

In reality, the distribution of most data is non-linear. In general, one solution for traditional machine learning algorithms to solve such problems is to map non-linear data to high-dimensional space into linear classification problems, but this method can lead to dimensional disasters and increased computational complexity. A kernel function is a function that satisfies the Formula 21.


(21) $$\begin{array}{l} K\left(x,z\right)=<\phi \left(x\right),\phi \left(z\right)> \end{array}$$

After introducing a small-scale mapping in a high-dimensional space for nonlinear problems, the original target equation is transformed into an Equation 22.


(22) $$\begin{array}{l} \underset{w,b}{\min}\frac{{\left\|w\right\|}^{2}}{2}+C\overset{N}{\underset{i=1}{\sum }}\xi_{i} \end{array}$$

In this target equation, the dimension *w* will become higher because the dimension ϕ(*x*_*i*_) becomes higher, resulting in an increase in calculation amount and even the inability to solve it. Therefore, the original objective equation is transformed into its dual form by introducing the Lagrange multiplier. As shown in Equations 23, 24.


(23) $$\begin{array}{l} \begin{array}{c} F=\underset{\alpha ,\beta ,\gamma }{\max}\frac{1}{2}\overset{N}{\underset{i=1}{\sum }}\overset{N}{\underset{j=1}{\sum }}y_{i}y_{j}\alpha_{i}\alpha_{j}K\left(x_{i},x_{j}\right)\\ +\frac{1}{2}\overset{u}{\underset{s=1}{\sum }}\overset{u}{\underset{t=1}{\sum }}\left(\beta_{s}-\gamma_{s}\right)\left(\beta_{t}-\gamma_{t}\right)K\left(x_{s}^{*},x_{t}^{*}\right)\\ +\overset{N}{\underset{i=1}{\sum }}\overset{u}{\underset{s=1}{\sum }}\alpha_{i}y_{i}\left(\beta_{s}-\gamma_{s}\right)K\left(x_{i}^{*},x_{s}^{*}\right)\\ -\overset{N}{\underset{i=1}{\sum }}y_{i}\alpha_{i}+\overset{u}{\underset{s=1}{\sum }}\beta_{s}-\overset{u}{\underset{s=1}{\sum }}\gamma_{s} \end{array} \end{array}$$

(24) $$\begin{array}{l} \overset{N}{\underset{i=1}{\sum }}y_{i}\alpha_{i}+\overset{u}{\underset{s=1}{\sum }}\beta_{s}+\overset{u}{\underset{s=1}{\sum }}\gamma_{s}=0\\ s.t.\;0\leq \alpha_{i}\leq C_{1},i=1,\ldots ,N\\ 0\leq \beta_{s},\gamma_{s}\leq C_{2},s=1,\ldots ,u \end{array}$$

Among them *K*(*x*_*i*_, *x*_*j*_) = < ϕ(*x*_*i*_), ϕ(*x*_*j*_)>. For the solution of the dual problem, the decision equation is shown in Formula 25.


(25) $$\begin{array}{l} f\left(x\right)=sgn\left(\alpha^{*}y_{i}K\left(x_{i},x+b^{*}\right)\right) \end{array}$$

This section introduces several common models and feature selection techniques of text emotion classification in emotion analysis, and also introduces the concept of conditional random model and word vector model, mathematical model, and key algorithm, and compares the previous algorithm with the new algorithm. Finally, the evaluation index of emotion analysis is introduced to deepen the understanding of emotion classification and the problem-solving method in the field of emotion analysis.

## Brand Evaluation Experiment Based on the Fusion Model

### Brand Evaluation Experiment

The test data set selected in the experiment is the review data of several cosmetics brand-related products sampled from Taboo review data, including Lancôme, Estee Lauder, Clinique, L'Oreal, Yuexin Wind Yin, Baibirds antelope, and other brands, 10,000 each. A total of 60,000 comments were marked according to the annotation rules and indicated whether the comment data were favorable or bad. The specific labeling work is responsible for the cooperative unit itself to ensure its accuracy and universality. The annotated data were divided into a training set and test set according to a ratio of 9:1. The data of the test set were sequence-annotated using the CRF model, and the named entities were extracted.

Based on prior knowledge and intuitive prediction, it is obvious that the design of an appropriate system can achieve more accurate results. In theory, supervised learning can be used to train the most suitable weight system. But doing so requires a lot of accurate supervision scores from different brands, which requires a lot of manpower and material resources. This paper optimizes the process of selecting the weight system iteratively, that is, first design several sets of systems, estimate the brand score, and then use the correct rate of the estimated score to evaluate whether the weight design is reasonable, select a more reasonable weight system to predict the scores of other brands, and evaluate the scoring results. This article takes cosmetics as an example and designs the following three sets of weight systems. To facilitate normalization, the total weight is 1. As shown in Tables 1–3.

**Table 1:** Cosmetics class weight system a.

**Entity classification**	**Grade**	**Weight**
Brand	2	0.3
Category	3	0.25
Effect	4	0.15
Pigment	5	0.1
Raw material	6	0.15
Shopping experience	9	0.05

**Table 2:** Cosmetic class weight system b.

**Entity classification**	**Grade**	**Weight**
Brand	2	0.167
Category	3	0.167
Effect	4	0.167
Pigment	5	0.167
Raw material	6	0.167
Shopping experience	9	0.167

**Table 3:** Cosmetic class weight system c.

**Entity classification**	**Grade**	**Weight**
Brand	2	0.1
Category	3	0.1
Effect	4	0.25
Pigment	5	0.25
Raw material	6	0.25
Shopping experience	9	0.05

The c-weight systems in Table 3 shows three cases. First, the weight is set according to the frequency of brand, category, efficacy, color, raw materials, shopping experience, etc. Second, some indicators have the same weight. Third, the attribute value is more important. This weight design is combined with the algorithm of the previous sections for the brand scoring experiment.

### Analysis of the Experimental Data

**Figure 1:**
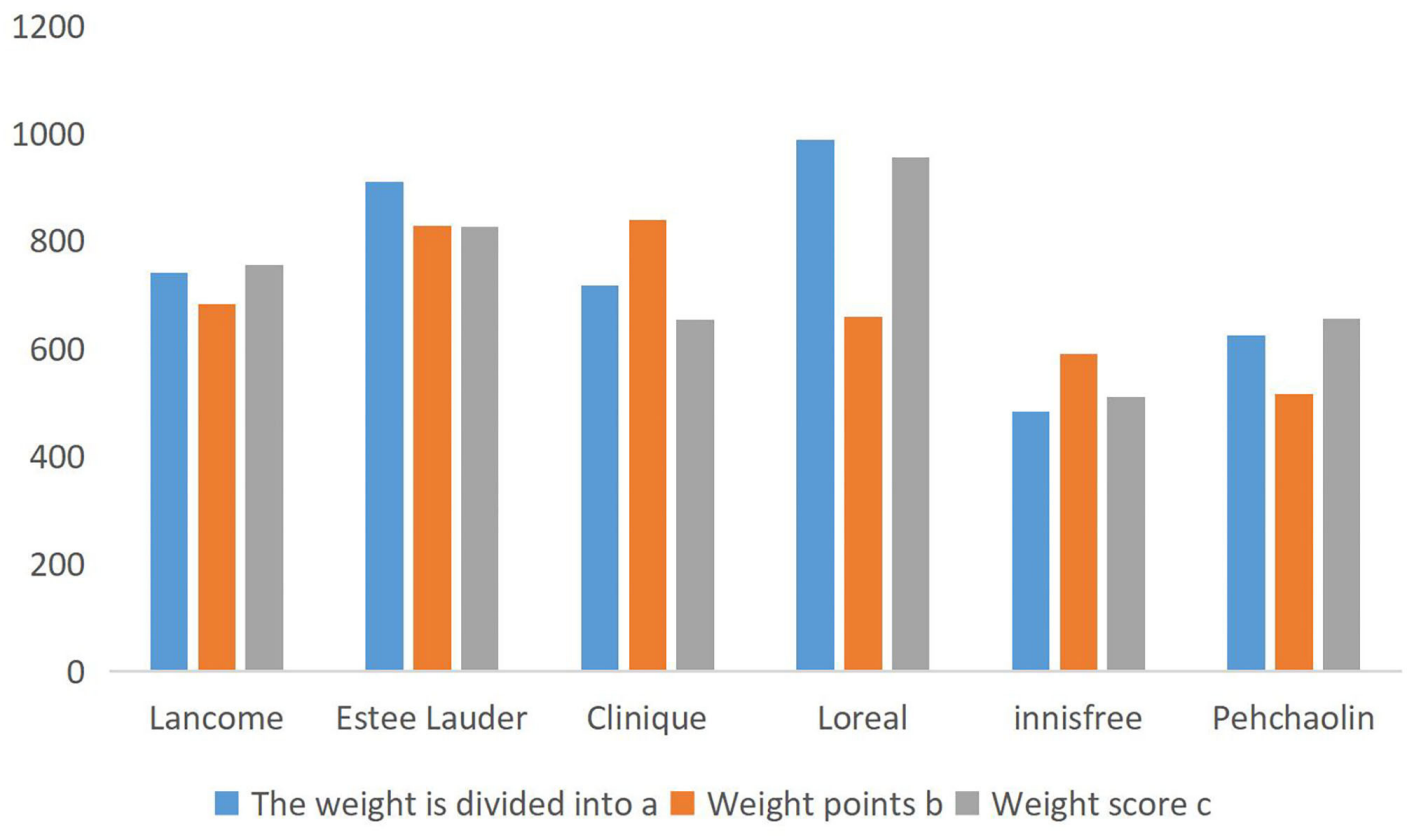
Comparison of the data scores of different brands.

For each brand, the identification results of emotion analysis using the CRF-DEEP algorithm are shown in Figure 1, still with 10,000 data per brand. The named entities, included in each piece of data, are not listed in detail. At the same time, the common scoring system that defines the brand evaluation is the number of good reviews minus the number of bad reviews.

For the very subjective objectivity of brand scoring, the industry has not thought of a very scientific and objective evaluation system, and the existing methods do not have reasonable evaluation indicators. Therefore, this paper takes the user questionnaire and ranking method to verify the results. For the above six brands, the questionnaire was used to analyze, and the target was women who used the six brands of cosmetics. By scoring these six brands, these brands are more popular among cosmetics brands, and 1 is set as the qualified score. The higher the number of brands used by users, the higher the scores, and the final results are shown in Table 4.

**Table 4:** The questionnaire scoring details of different brands.

**Brand**	**1 Points**	**Two points**	**Three points**	**4 Points**	**5 Points**	**Average**
Lancôme	86	118	343	278	175	834
Estee Lauder	135	82	178	416	189	860
Clinique	73	147	387	231	163	816
L'Oreal	65	82	256	244	241	916
Innisfree	282	372	154	49	143	599
Pehchaolin	194	473	127	62	144	622

For the actual research data, it can be seen that the weighted evaluation system based on emotion analysis proposed in this paper has a better matching degree.

**Figure 2:**
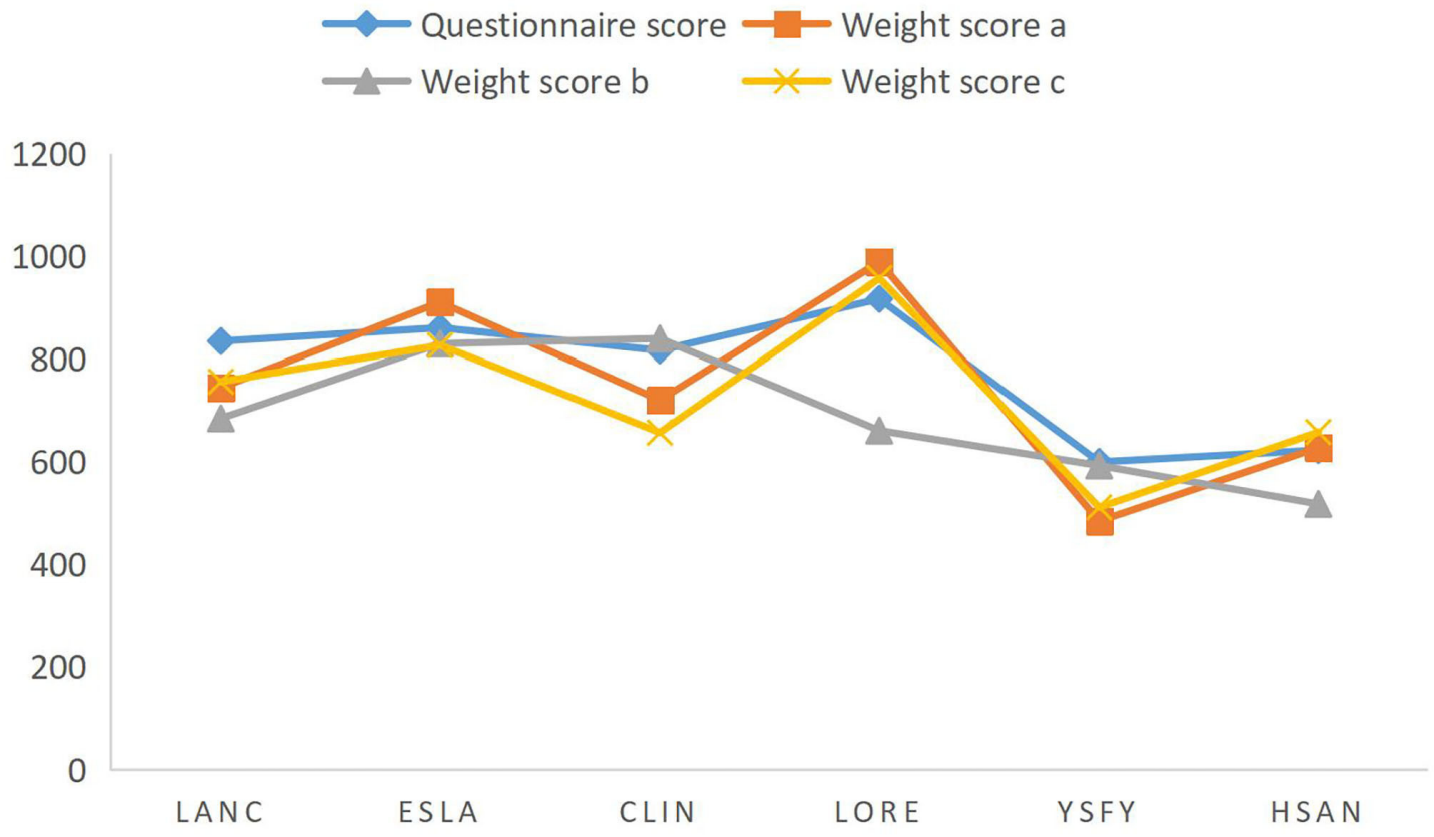
Brand score comparison chart.

Table 4 has been normalized to be within 1,000, and the scores of different brands shown in Figure 2 are drawn. By comparison, the brand score calculated by the weight score A algorithm has the highest fit with the user questionnaire, and the brand score effect obtained by weight C is slightly worse than weight A, while the brand score calculated by weight b is the worst. Although the weight score A is higher than B and C, it does not prove that the weight score A is a reasonable weight system globally, and it can be further found by using a genetic algorithm.

Genetic algorithm (Genetic Algorithm, GA), proposed by the American John holland in the 1970's, borrowed from Darwin's evolution and Mendel's genetics, is the natural selection and genetics of biological evolution model, its essence is an efficient, parallel, global search method, can automatically acquire and accumulate knowledge about the search space, and adaptively control the search process to get the best solution. As a random optimization and search method, it is characterized by directly operating the structure object with no limit of guidance and function continuity, parallel and better global optimization ability; probabilistic optimization method can automatically obtain and guide the optimized search space, and adjust the direction of search method without certain rules. The final optimal weight system results obtained by the genetic algorithm are shown in Table 5.

**Table 5:** Final weight system of cosmetics category.

**Entity classification**	**Grade**	**Weight**
Brand	2	0.272
Category	3	0.264
Effect	4	0.154
Pigment	5	0.119
Raw material	6	0.129
Shopping experience	9	0.062

Table 5 shows that the CRF model is more effective through 10-fold cross-validation, and the accuracy rate is the optimal parameter in the calculation process.

### Data Description

A brand contains many products and many models. Different types of goods have a large number of customers on the e-commerce platform, so customers generate a large amount of comment data. The quality of comments can represent the reputation of brands to some extent. According to the statistical principle, under the background of big data statistics, the user reputation can be regarded as a standard of brand quality.

**Figure 3:**
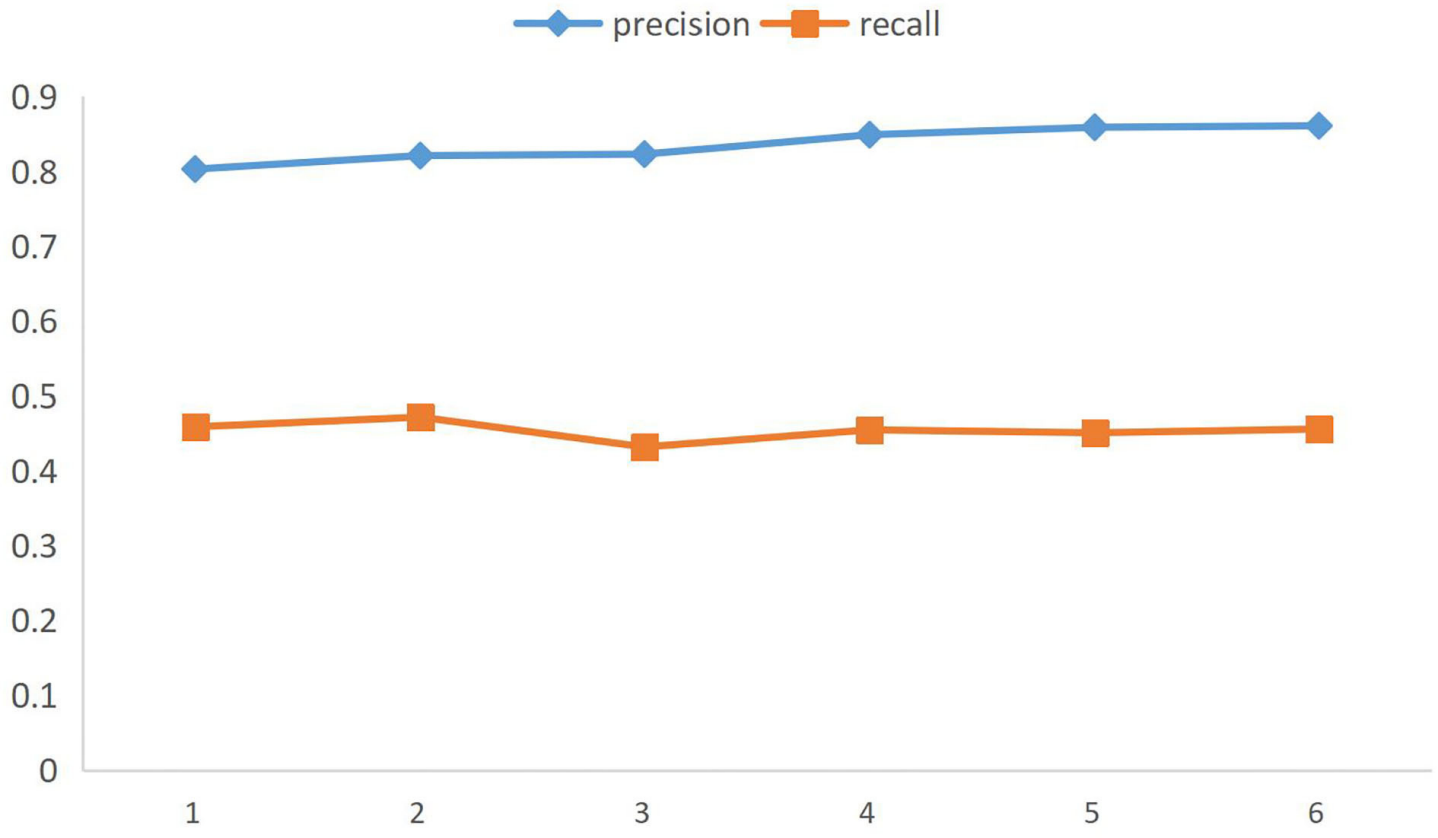
Figure of CRF model with brand data quantity.

However, we should not simply use the high praise proportion of all the products of a brand as the quality evaluation standard. Through big data, we can find that there is a lot of water army and shopping experience evaluation in the comments of online e-commerce, not simply for the quality of product. For example, the evaluation of “express delivery” and “customer service attitude” is obviously due to the uneven quality of the seller itself, rather than the quality of the product itself. Therefore, this paper uses named entities to depict the core meaning of network comments, to accurately distinguish the subjects of user evaluation and more accurately evaluate the quality of e-commerce brands. To validate the actual recognition and generalization ability of the CRF model, the training set and test set are expanded below to combine multiple brand data for training tests. The comparative test map is shown in Figure 3, and the test results are shown in Table 6.

**Table 6:** Changes of the model along with the quantity of brand data.

**Brand number**	**Precision**	**Recall**
1	0.803	0.459
2	0.821	0.472
3	0.823	0.432
4	0.849	0.455
5	0.859	0.451
6	0.861	0.456

Figure 3 is a CRF model test done with data aggregated from any of the six brands, respectively. It can be seen that in the case of more and more data and more complex data, the recall rate decreases slightly and the accuracy increases steadily. It can be seen that the CRF model has a strong generalization ability and is suitable for application in big data. It is worth noting that the selected brands all belong to the big category of cosmetics, and the data has a certain similarity, which is suitable for the same model training. In other tests, the data gap between different major categories is huge, and exclusive annotation rules and template models need to be customized, but the basic ideas are the same. From the results, the named entity identification results of this model reach the advanced level. So, based on the work of named entity identification, a new classification model will be introduced here to conduct emotion identification and, finally, achieve the purpose of brand evaluation. Accuracy and recall are two measures widely used in information retrieval and statistical classification, which are used to evaluate the quality of results. Precision is the ratio between the number of relevant documents retrieved and the total number of documents retrieved, which measures the precision of the retrieval system; Recall rate refers to the ratio between the number of relevant documents retrieved and the number of all relevant documents in the document library, which measures the recall rate of the retrieval system.

Here, the annotation data of six brands will be further selected for analysis, and the comment data collected from e-commerce websites are manually identified with positive evaluation marks. The absence of the evaluation system for e-commerce websites themselves is because there are a large number of favorable comments. Therefore, CRF annotation and artificial emotion annotation can enhance the effectiveness of the data. The total data volume is 60,000, each brand data volume is 10,000, and the training set and test set are randomly generated in a ratio of 9:1. In Table 7, the different methods for the accuracy of brand data classification, respectively, for the conditional random airport extraction features and support vector machine classification combination, conditional random airport extraction features and logical regression classification combination, information gain extraction features and support vector machine classification, support vector machine classification four combinations of accuracy results data are given:

**Table 7:** The accuracy comparison of the data from different brands.

**Brand**	**CRF-SVM**	**CRF-LR**	**IG-SVM**
Lancôme	0.92	0.88	0.76
Estee Lauder	0.90	0.89	0.78
Clinique	0.87	0.85	0.72
L'Oreal	0.89	0.91	0.79
Innisfree	0.83	0.84	0.81
Pehchaolin	0.84	0.86	0.77

**Figure 4:**
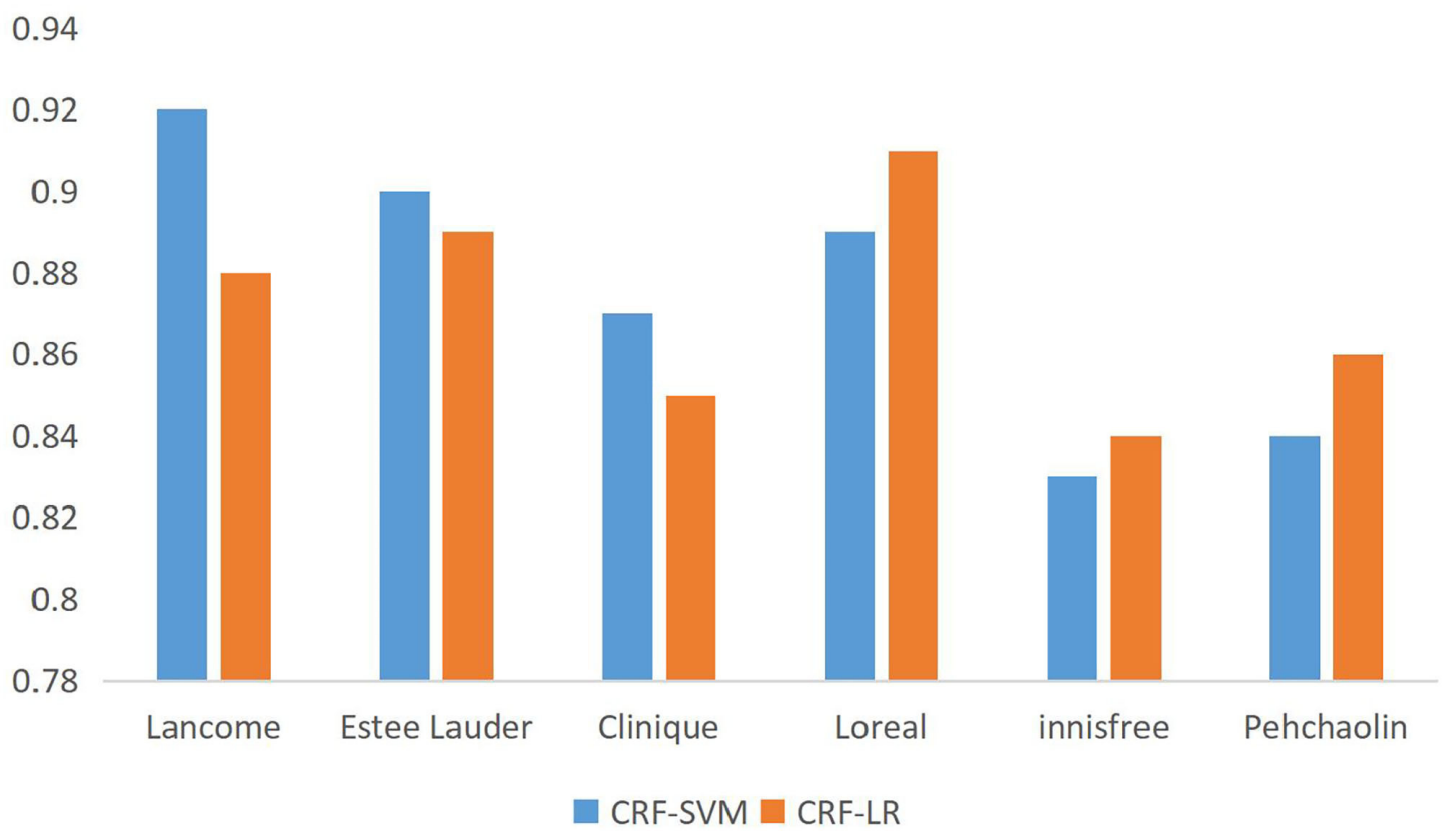
Precision rate comparison of data from different brands.

A comparative map of the accuracy of the different brand data can be obtained from Table 7, as shown in Figure 4.

**Figure 5:**
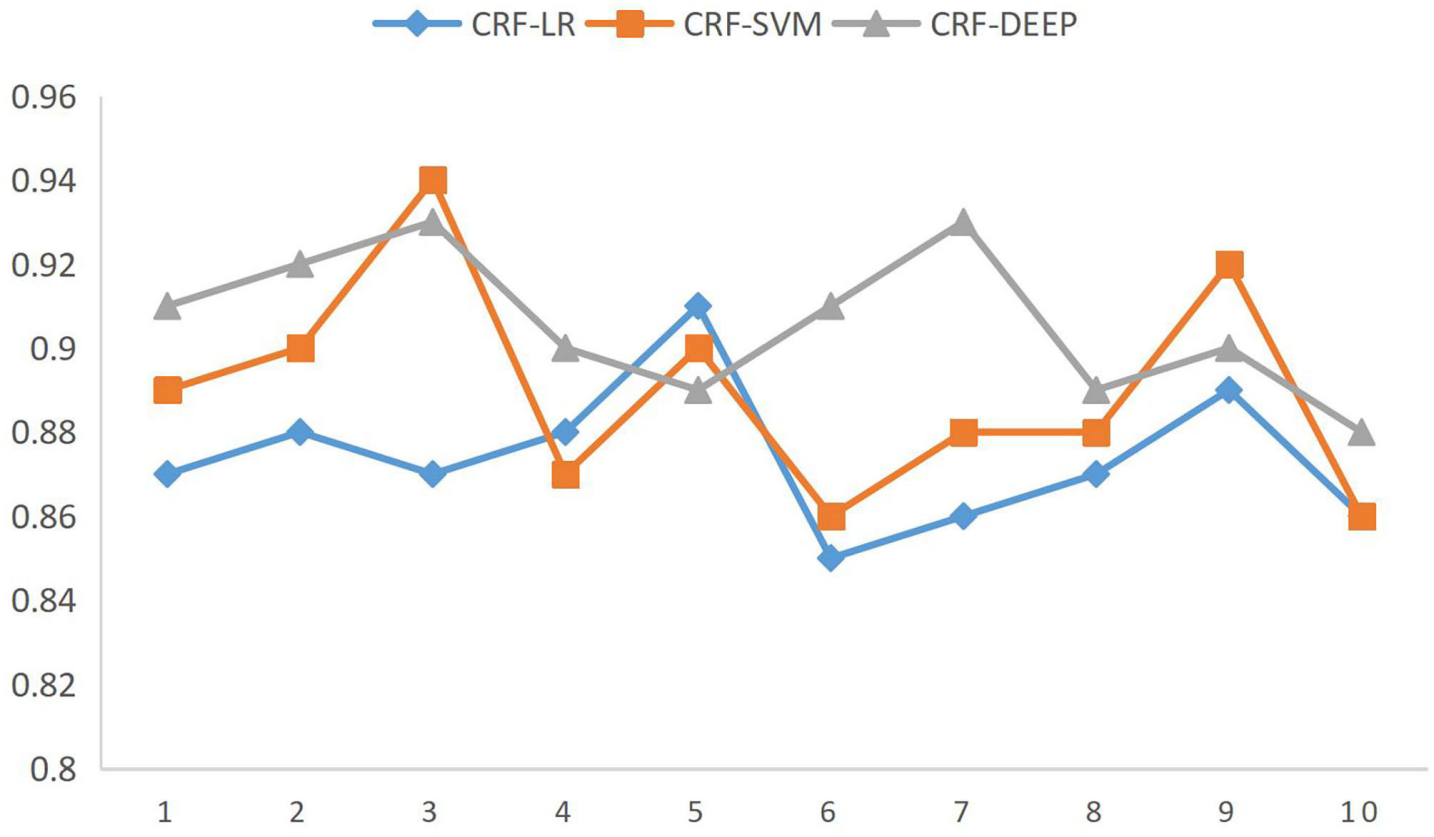
Ten-fold cross-validation accuracy comparison figure.

The accuracy comparison in Table 7 shows that the algorithm based on CRF-SVM proposed above has the best results. At the same time, it is found that there is no significant difference between the classifier of support vector machine or logistic regression and the feature extraction algorithm of information gain or CRF named entity. Through the analysis of the data, we can get the reason, for natural language, that the expression methods are strange, especially when Chinese has the same word that does not agree, different sentence patterns is not too rigorous grammar, and the choice of characteristics is particularly important. It can be said that for the Chinese natural language expression, the choice of the correct characteristics can be twice the result with half the effort. To verify the scalability of the algorithm to fit the data, the full data were fused again, and the accuracy was compared by the 10-fold cross-validation method, as shown in Figure 5.

The ordinate represents the accuracy rate, which is obtained by comparing the three brand data with the actual data through the CRF-SVM algorithm.

### Experimental Results

The experiment first introduces the evaluation system of commodities, then introduces the use method of CRF models and tools, extracts the named entities using custom feature optimization, compares the experimental results, and proves that the feature template designed in this paper has good accuracy and recall index. Then expounds the optimization scheme of two emotion classification algorithms designed in this paper—classification method considering named entities and incorporating deep features. Both algorithms consider more hidden information in the data, complementing the classification algorithm features from the perspectives of named entity and word vector, respectively. Comparing these two algorithms and baseline algorithms, respectively, we prove that the model method proposed in this paper has some optimization in real data. Finally, we propose a new definition system for brand evaluation to define the brand quality from the resulting design weight division of emotion recognition.

## Conclusion

This paper focuses on the commercial brand evaluation method based on multi-feature fusion emotion analysis and intends to apply the method to future business. The method of brand evaluation is divided into two subtasks: feature extraction and emotion classification. In the feature extraction subtask, the features extracted by the two methods of identifying named entities and multi-feature fusion were input into the classifier. Compared with the output results of the classifier, the experimental data showed that the feature files generated by the multi-feature fusion shallow and deep feature fusion methods were input into the classifier, indicating the higher accuracy of the classification results. The feature fusion method is to sum and normalize the deep-named entity identified by the conditional random field model and the word vector model, extract two sets of feature vectors with the same pattern, establish the correlation criterion function between the two sets of feature vectors, and obtain a new feature set. In addition, we compared two classification models of logistic regression and SVM in the emotion classification subtask and verified the real data; the experimental results showed that the accuracy of classification results using the SVM model is higher. Finally, the experiment compares the three brand evaluation methods. The experimental data shows that the classification accuracy of the third method is higher, and this method is applied to the brand research in the paper, which is more helpful to quantify users' views and attitudes toward the brand. There will be new ideas on the road of scientific research, and the optimization of feature extraction methods still needs to be explored continuously. In this paper, the part-of-speech tagging in feature extraction adopts the manual tagging method, which is still limited, inconvenient to expand and copy to other fields, and relies on manual rather than a machine to work. At the same time, deep feature extraction not only ensures the diversity of features but also increases the complexity of computation. Therefore, in future research work, I will try my best to find a compromised feature extraction method to make the feature extraction technology more excellent.

## Data Availability Statement

The original contributions presented in the study are included in the article/supplementary material, further inquiries can be directed to the corresponding author.

## Ethics Statement

Ethical review and approval was not required for the study on human participants in accordance with the local legislation and institutional requirements. Written informed consent from the patients/participants or patients/participants legal guardian/next of kin was not required to participate in this study in accordance with the national legislation and the institutional requirements.

## Author Contributions

The author confirms being the sole contributor of this work and has approved it for publication.

## Conflict of Interest

The author declares that the research was conducted in the absence of any commercial or financial relationships that could be construed as a potential conflict of interest.

## Publisher's Note

All claims expressed in this article are solely those of the authors and do not necessarily represent those of their affiliated organizations, or those of the publisher, the editors and the reviewers. Any product that may be evaluated in this article, or claim that may be made by its manufacturer, is not guaranteed or endorsed by the publisher.
